# Canine and human gastrointestinal stromal tumors display similar mutations in *c-KIT* exon 11

**DOI:** 10.1186/1471-2407-10-559

**Published:** 2010-10-15

**Authors:** Emmalena Gregory-Bryson, Elizabeth Bartlett, Matti Kiupel, Schantel Hayes, Vilma Yuzbasiyan-Gurkan

**Affiliations:** 1 Comparative Medicine and Integrative Biology Program, College of Veterinary Medicine, Michigan State University, East Lansing, Michigan, USA; 2 Department of Microbiology and Molecular Genetics, Michigan State University, 2209 Biomedical and Physical Sciences, East Lansing, Michigan, USA; 3 Department of Pathobiology and Diagnostic Investigation, Diagnostic Center for Population and Animal Health, Michigan State University, 4125 Beaumont Road, Lansing, Michigan, USA; 4 Department Small Animal Clinical Sciences, College of Veterinary Medicine, Michigan State University, East Lansing, Michigan, USA

## Abstract

**Background:**

Gastrointestinal stromal tumors (GISTs) are common mesenchymal neoplasms in the gastrointestinal tract of humans and dogs. Little is known about the pathogenesis of these tumors. This study evaluated the role of *c-KIT *in canine GISTs; specifically, we investigated activating mutations in exons 8, 9, 11, 13, and 17 of *c-KIT *and exons 12, 14, and 18 of platelet-derived growth factor receptor, alpha polypeptide (*PDGFRA*), all of which have been implicated in human GISTs.

**Methods:**

Seventeen canine GISTs all confirmed to be positive for KIT immunostaining were studied. Exons 8, 9, 11, 13 and 17 of *c-KIT *and exons 12, 14, and 18 of *PDGFRA*, were amplified from DNA isolated from formalin-fixed paraffin-embedded samples.

**Results:**

Of these seventeen cases, six amplicons of exon 11 of *c-KIT *showed aberrant bands on gel electrophoresis. Sequencing of these amplicons revealed heterozygous in-frame deletions in six cases. The mutations include two different but overlapping six base pair deletions. Exons 8, 9, 13, and 17 of *c-KIT *and exons 12, 14, and 18 of *PDGFRA *had no abnormalities detected by electrophoresis and sequencing did not reveal any mutations, other than synonymous single nucleotide polymorphisms (SNPs) found in exon 11 of *c-KIT *and exons 12 and 14 of *PDGFRA*.

**Conclusions:**

The deletion mutations detected in canine GISTs are similar to those previously found in the juxtamembrane domain of *c-KIT *in canine cutaneous mast cell tumors in our laboratory as well as to those reported in human GISTs. Interestingly, none of the other *c-KIT *or *PDGFRA *exons showed any abnormalities in our cases. This finding underlines the critical importance of *c-KIT *in the pathophysiology of canine GISTs. The expression of KIT and the identification of these activating mutations in *c-KIT *implicate KIT in the pathogenesis of these tumors. Our results indicate that mutations in *c-KIT *may be of prognostic significance and that targeting KIT may be a rational approach to treatment of these malignant tumors. This study further demonstrates that spontaneously occurring canine GISTs share molecular features with human GISTs and are an appropriate model for human GISTs.

## Background

**G**astro**i**ntestinal **s**tromal **t**umors (GISTs) are one of the most common mesenchymal tumors that arise from the wall of the gastrointestinal tract. Gastrointestinal stromal tumors occur in many species including humans, dogs [1-3], and horses [4,5]. GISTs can metastasize to the liver and peritoneal cavity, warranting a very poor prognosis. In humans, approximately 70% of GISTs occur in the stomach and 20% occur in the small intestine [6,7], whereas in dogs the reverse is true with 76% of GISTs occurring in the small intestine and colon, while 19% occur in the stomach [2].

The majority of GISTs are diagnosed by the demonstration of the expression of KIT (CD117), a type III tyrosine kinase receptor encoded by the proto-oncogene *c-KIT *[2,8-11]; although a small proportion of GISTs do not exhibit CD117 immunoreactivity [12]. KIT has critical roles in cell differentiation, proliferation and migration, especially in hematopoietic, neural crest, and germ cell lineages [13]. In addition, KIT along with its ligand, stem cell factor (SCF), also known as steel factor, is necessary for the development of melanocytes, mast cells, and interstitial cells of Cajal [14]. It has been suggested that GISTs may originate from the interstitial cells of Cajal, which are pacemaker cells responsible for regulating peristalsis in the gastrointestinal tract [15,16].

The KIT receptor is a cell surface receptor consisting of an extracellular domain, a transmembrane domain, and a cytoplasmic domain, which includes the juxtamembrane and kinase domains [17,18]. The juxtamembrane domain (amino acid residues 543-580) [13] is a highly conserved region of KIT located between the transmembrane domain (amino acid residues 521-543) [17] and kinase domain (amino acid residues 581-936) [13]. The KIT juxtamembrane domain is primarily coded for by exon 11 of *c-KIT*, while the split kinase domain are coded for by exons 12-18 of *c-KIT *[19]. The juxtamembrane domain regulates the enzymatic activity of KIT by preventing relative movement of the protein and thus inhibiting receptor dimerization [20]. In normal cells, binding of the SCF ligand to the KIT receptor results in receptor homodimerization and subsequent activation of the KIT receptor via cross phosphorylation of tyrosine residues on the opposite KIT homodimer partner [20]. The phosphotyrosines become binding sites and activators of several cell-signaling proteins including JAK2 and PI3K [21]. These particular proteins are the start of the JAK-STAT and JNK pathways, leading to a potent intracellular signal for the cell to proliferate [21,22]. Gain-of-function mutations in KIT among human GISTs have demonstrated that the constitutive activation of KIT in the absence of its ligand and without dimerization may play a critical role in GIST tumorigenesis [23].

In humans, mutations in *c-KIT *have been reported in more than 65% of GIST cases [8,20,23,24], and in GISTs with wild-type *c-KIT*, mutations of platelet-derived growth factor receptor, alpha polypeptide (*PDGFRA*) were found in 35% of those cases [25]. *PDGFRA *codes for a transmembrane type III tyrosine kinase receptor for members of the platelet-derived growth factor family, which are mitogens for cells of mesenchymal origin. GISTs with *c-KIT *or *PDGFRA *mutations have similar downstream signaling pathways, suggesting that *PDGFRA *mutations serve as an alternative tumorigenic mechanism to *c-KIT *in GISTs [25]. Mutations have been found in exons 11, 9, 13, and 17 of *c-KIT *in sporadic GISTs, with exon 11, the juxtamembrane domain, being the most frequent site of mutations [16,26,27], comprising up to 90% of all *c-KIT *mutations [28]. Exon 8 of *c-KIT *has been reported to have mutations in other types of neoplasias. Most GISTs are sporadic, but familial GIST syndromes presenting with multiple GISTs have been reported in humans [29]. Affected family members often harbor germline mutations of the *c-KIT *gene in their tumors and leukocytes [29,30] and there is a report of one family with a germline mutation in *PDGFRA *[31]. The familial GIST syndrome has been recapitulated in two knock-in mouse models, one designed with a V558 deletion mutation in exon 11 of *c-KIT*, and the other carrying a K-to-E amino acid mutation at position 641 in exon 13 of *c-KIT *[32,33].

**Figure 1:**
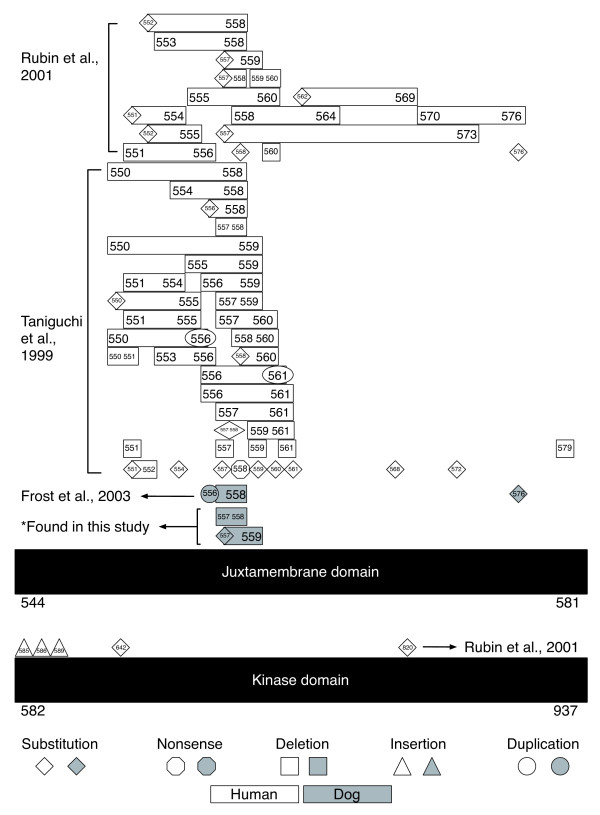
**Comparison of *c-KIT* mutations in human and canine GISTs**. Human mutations are in white [8,9,21]; dog mutations are indicated in gray [2,3]. Codon numbering is based upon the human amino acid sequence [GenBank: NP_000213]. The asterisk indicates the two deletion mutations found in this study.

All of the reported mutations in *c-KIT *could potentially lead to the activation of KIT in the absence of its ligand. Constitutively activated KIT would then give rise to the development and/or progression of gastrointestinal stromal tumors in dogs. A comparison of the currently documented mutations found in *c-KIT *in humans and canines is presented in Figure 1. The reported canine *c-KIT *mutations have been associated with mast cell tumors [34,35] as well as GISTs [2]. The purpose of this study was to evaluate the role of *c-KIT *and *PDGFRA *in canine GISTs. While KIT immunopositivity has been demonstrated in canine GISTs in two previous studies [2,3], only the study by Frost et al. has explored mutations in *c-KIT *to date, where four archived canine GISTs were examined, revealing mutations in exon 11 of *c-KIT *in two of the cases. Our present study investigates exons 8, 9, 11, 13, and 17 of *c-KIT *and exons 12, 14, and 18 of *PDGFRA *for mutations in a larger sample set of canine GISTs, providing information on *c-KIT *mutational status in seventeen cases.

## Methods

### Cases

**Figure 2:**
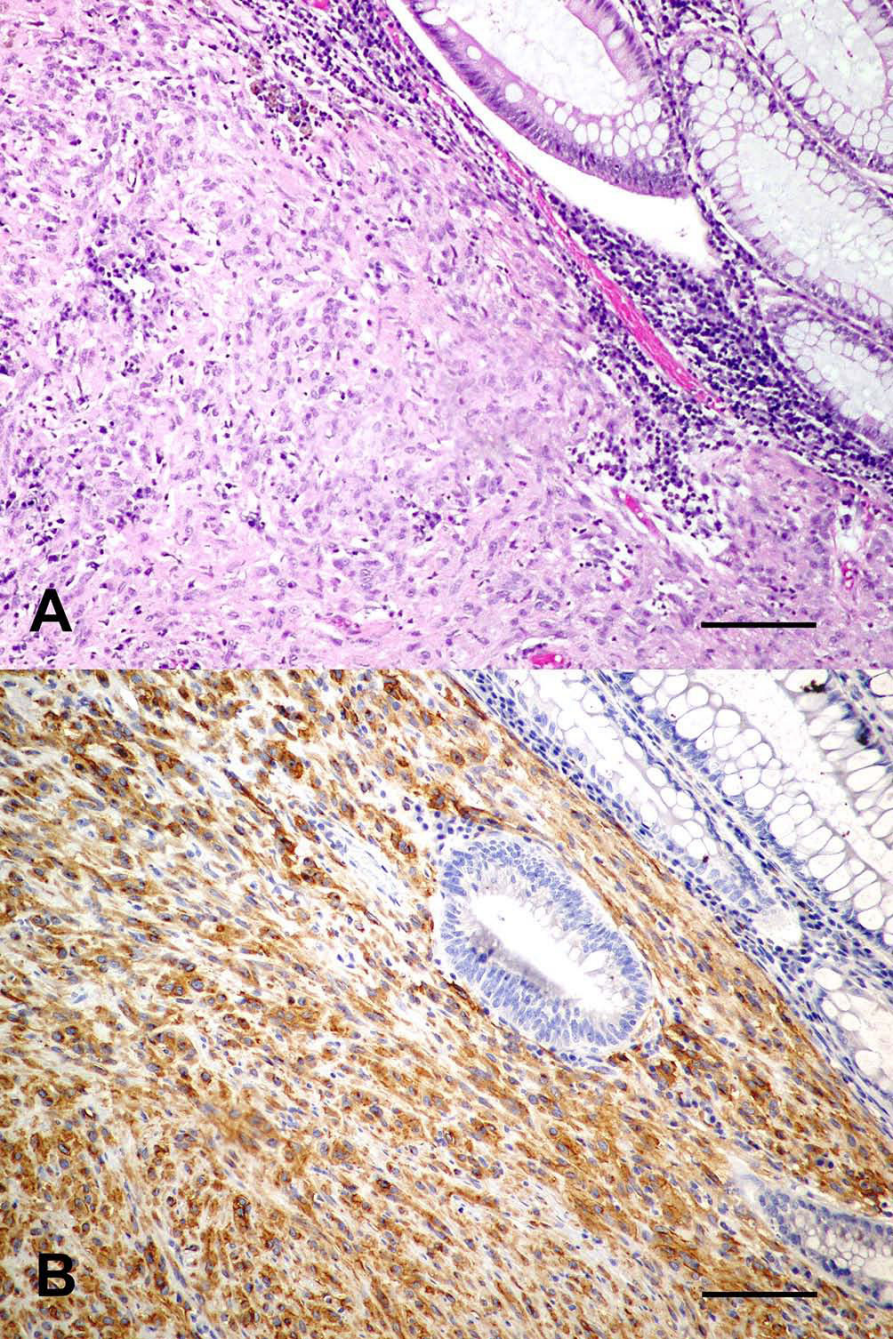
**GIST**. 2a - Section of a gastrointestinal stromal tumor analyzed in this study stained with hematoxylin and eosin. 2b - The same gastrointestinal stromal tumor with immunohistochemical staining showing abundant KIT expression as reflected by the brown deposits.

Forty-six cases of canine gastrointestinal smooth muscle tumors were submitted to the Michigan State University Diagnostic Center for Population and Animal Health from 1991 to 2006. All tumors originated from surgical biopsies that were immediately fixed in 10% neutral buffered formalin and embedded in paraffin within 24-48 hours, following routine protocols. From this pool of cases eighteen tumors were diagnosed as GISTs confirmed by characteristic histomorphology and positive KIT staining by immunohistochemistry and were included in this study (Figures 2a and 2b). The age of the dogs in this study ranged from 4 to 15 years with a mean age of 10.9 years. Various purebred and mixed bred dogs were included with a gender ratio of 72% female to 28% male dogs. Tumor sites were distributed throughout the gastrointestinal tract from the stomach to the cecum as indicated in Table 1. A histologically normal, non-neoplastic tissue sample from each dog was also analyzed to determine the *c-KIT *mutation status in constitutive DNA.

**Table 1:** Samples

Case	Breed	Age	Sex	Tumor Site	*c-KIT *Mutations	*PDGFRA *Mutations
1	Cockapoo	13	FS	stomach	exon 11 deletion, exon 11 SNP	normal

2	German Wirehaired Pointer	10.5	FS	cecum	exon 11 deletion	exon 12 SNP, exon 14 SNP

3	Cocker Spaniel	12	FS	small intestine	normal	normal

4	Labrador Retriever	4	FS	duodenum	normal	normal

5	American Pit Bull Terrier	15	FS	cecum	exon 11 deletion	normal

6	Golden Retriever	13	F	small intestine	normal	normal

7	Collie	12	M	jejunum	exon 11 SNP	normal

8	Boxer	10	M	jejunum	normal	normal

9	Cocker Spaniel	US	FS	small intestine	exon 11 SNP	normal

10	Irish Setter	11	FS	small intestine	normal	normal

11	Golden Retriever	10.5	MN	cecum	exon 11 SNP	normal

12	Springer Spaniel	10.6	FS	cecum	exon 11 deletion	normal

13	Labrador Retriever	8.5	FS	jejunum	normal	normal

14	German Shorthaired Pointer	9	FS	small intestine	normal	exon 12 SNP

15	Labrador Retriever cross	10	MN	ileocecocolic junction	exon 11 deletion	normal

16	Mixed	9	FS	cecum	exon 11 deletion	exon 12 SNP, exon 14 SNP

17	Mixed	15	MN	jejunum	normal	normal

Signalment and site of canine gastrointestinal stromal tumor cases analyzed in this study. FS = female spayed, MN = male neutered, US = unspecified.

### DNA isolation from formalin- fixed paraffin-embedded (FFPE) sections

Neoplastic tissue, less than 1 mm^3^, was excised from each FFPE block to retrieve sections corresponding to KIT positive immunostaining areas. Similarly, sections of histologically normal tissue, negative for KIT immunostaining were also collected from each case. From these tissue sections DNA was isolated as described previously [34,36]. The tissue section was placed in 400 *μ*l of digestion buffer (50 mM Tris, pH 8.5, 1 mM ethylenediaminetetraacetic acid [EDTA], 0.5% Tween) and heated to 95°C for 10 minutes to melt the paraffin. The tissue solutions were then subjected to high power microwave irradiation twice for 30 seconds each with vortexing after each heating step. After cooling, 5 *μ*l of 15 mg/ml proteinase K was added to each solution and incubated overnight at 42°C. Following protein digestion, proteinase K was inactivated at 95°C for 10 minutes. The solutions were then centrifuged and 150 *μ*l was aliquoted to be used as DNA template in subsequent polymerase chain reaction (PCR).

### Amplification of *c-KIT* juxtamembrane and kinase domains

Exon 11, coding for the juxtamembrane domain of KIT, and exon 17, coding for the kinase domain of KIT, were amplified from these tissue sections via PCR using primers and conditions optimized in earlier studies [37,38]. Exon 8, 9, and 13 of *c-KIT *and exons 12, 14, and 18 of *PDGFRA *were also amplified (Table 2). The PCRs were set-up in 25 *μ*l total reaction volume consisting of 50 ng of DNA template prepared as described above: 5 pmol of each primer, 0.5 U of Taq polymerase (Invitrogen, Carlsbad, CA) and final concentrations of 80 *μ*M deoxynucleoside triphosphate, and 2 mM MgCl_2_. Cycling conditions for the PCR were 94°C for 4 minutes; 40 cycles of 94°C for 1 minute, annealing temperatures averaging 58°C for 1 minute, and 72°C for 1 minute; followed by a final elongation step at 72°C for 5 minutes. PCR products were then subjected to electrophoresis on 2% agarose gels and visualized under ultraviolet light after ethidium bromide staining.

**Table 2:** Primer sets

Amplified Region	Forward	Reverse
*c-KIT *exon 8	5'-CAGCAGTCTGACCTATGGC -3'	5'-GCTCAGCTCCTGGACAGAAA-3'

*c-KIT *exon 9	5'-GATTGATTGATTGATTTTCCTAG-3'	5'-GCAGGCAGAGCCTAAACATC-3'

*c-KIT *exon 11	5'-CATTTGTTCTCTACCCTAAGTGCT-3'	5'-GTTCCCTAAAGTCATTGTTACACG-3'

*c-KIT *exon 13	5'-CTGATTAAGTCGGATGCGGC-3'	5'-CAAGCACTGTCGCAATGG-3'

*c-KIT *exon 17	5'-ATAGCAGCATTCTCGTGTTG-3'	5'-AACTAAAATCCTTCACTGGACTG-3'

*PDGFRA *exon 12	5'-TTAATGGCTCTGATTGCTCAC-3'	5'-CACCCAGTGCTCATAACCTC-3'

*PDGFRA *exon 14	5'-ACTGGTTTTGGTTCCCACAG -3'	5'-CAATGATTCGCAGCAACG-3'

*PDGFRA *exon 18	5'-TAGCTCAGCCGTGGGTATG-3'	5'-CACATGAGCAGAGATGTCAGG-3'

Primer sets used for the amplification of the indicated exons of *c-KIT *and *PDGFRA *during PCR.

### Sequencing

Amplified fragments from all tissue sections were characterized by automated sequencing. The PCR product for each section was submitted in 5 *μ*l quantities with 30 pmol of the appropriate primer to Michigan State University's Genomics Technology Support Facility. This facility utilizes the automated direct sequencing technique, which incorporates fluorescently labeled dideoxynucleotides during cycle sequencing and separates the resulting products by capillary electrophoresis for detection on an ABI 3700 sequence analyzer (Foster City, CA).

## Results

Of the eighteen KIT immunopositive cases, seventeen cases yielded amplification products. The remaining case did not yield amplification products with any of the *c-KIT *or *PDGFRA *primer sets or with primers for unrelated canine genes.

**Figure 3:**
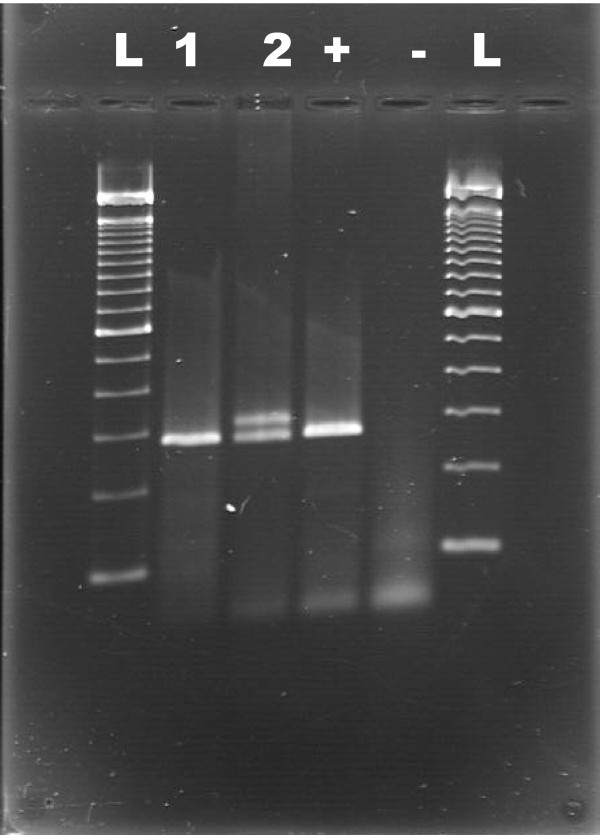
**Electrophoresis of PCR product of exon 11 of *c-KIT*, juxtamembrane domain**. L = 100 bp ladder; 1 = case with normal exon 11 of *c-KIT*, confirmed by sequencing; 2 = case with aberrant banding pattern; + = positive PCR control (normal dog spleen); - = negative PCR control (water)

**Figure 4:**
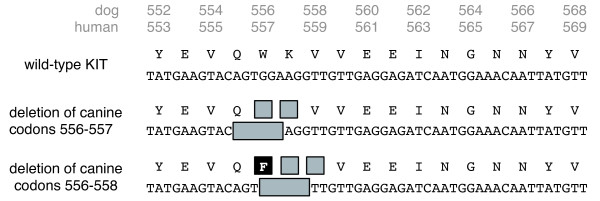
**Deletion mutations found in exon 11 of *c-KIT***. Sequence analysis results illustrating the two deletion mutations present in the six cases with the aberrant banding pattern. This region of exon 11 of canine *c-KIT* [GenBank: NP_001003181] differs by only one codon from humans [GenBank: NP_000213].

For exon 11 of *c-KIT*, six of these seventeen cases of canine GISTs displayed an aberrant banding pattern upon gel electrophoresis of the PCR product (Figure 3). The remaining eleven cases displayed a band similar to the positive control on electrophoresis, and analysis confirmed the sequence was identical to the wild-type exon 11 of *c-KIT *except for a single nucleotide polymorphism (SNP) located at base pair 50110905 C > T [GenBank: NC_006595.2] detected in four cases. However, sequence analysis of the aberrant six cases uncovered a mixture of normal and mutant alleles. Further examination identified short in-frame deletions (Figure 4). The mutations included two different, but overlapping 6 base pair deletions, which translated to a deletion of two amino acids in two of the cases and an amino acid change and a deletion of two amino acids in the other four cases. The first mutation (canine codons 556-557) occurred in two of the cases and consisted of the deletion of the sequence AGTGGA located at base pairs 50110838 to 50110843 of the canine genomic DNA [GenBank: NC_006595.2]. This mutation translated to a deletion of two amino acids, tryptophan and lysine, at codons 556 and 557 of canine KIT, respectively [GenBank: NP_001003181]. The second mutation (canine codons 556-558) was discovered in four of the cases and results in the deletion of the sequence GGAAGG located at base pairs 50110841 to 50110846 of the canine genomic DNA [GenBank: NC_006595.2]. This second mutation translated to a deletion of two amino acids, lysine and valine, at codons 557 and 558 of canine KIT, respectively [GenBank: NP_001003181]. The deletion of the last two guanines of the codon 556 in this mutation combined with deletion of the next 4 nucleotides resulted in an amino acid change from the tryptophan at codon 556 to a phenylalanine (Table 3). In these six cases, analysis of the normal tissue obtained from these dogs revealed sequences that were identical to the wild-type exon 11 of *c-KIT.*

**Table 3:** Deletion mutations found in canine GISTs in this study

Case	Codon	DNA Mutation	Amino Acid
2	556-557	GTA CAG TGG AAG GTT GTT → GTA C_AG GTT GTT	VQWKVV → VQVV

16	556-557	GTA CAG TGG AAG GTT GTT → GTA C_AG GTT GTT	VQWKVV → VQVV

1	556-558	GTA CAG TGG AAG GTT GTT → GTA CAG T TT GTT	VQWKVV → VQFV

5	556-558	GTA CAG TGG AAG GTT GTT → GTA CAG T TT GTT	VQWKVV → VQFV

12	556-558	GTA CAG TGG AAG GTT GTT → GTA CAG T TT GTT	VQWKVV → VQFV

15	556-558	GTA CAG TGG AAG GTT GTT → GTA CAG T TT GTT	VQWKVV → VQFV

The codon numbering is based upon the canine KIT amino acid sequence. The genomic DNA mutations were experimentally determined by direct sequencing and the altered sequence is underlined in column three. The expected amino acid changes in the protein are provided in column four. [GenBank: NC_006595.2, GenBank: NP_001003181].

**Figure 5:**
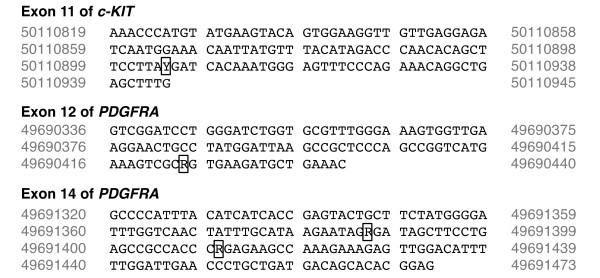
**Single nucleotide polymorphisms (SNPs)**. SNPs, demarcated by the boxed letters, found in exon 11 of *c-KIT *and exons 12 and 14 of *PDGFRA *[GenBank: NC_0006595.2] in the canine GIST samples (R = A or G; Y = C or T).

All seventeen cases were also amplified for exons 8, 9, 13, and 17 of *c-KIT*. Only the expected single band, similar to the positive control, was observed after gel electrophoresis. Sequencing of all PCR products obtained revealed no mutations in these GIST samples for exons 8, 9, 13, or 17 of *c-KIT*. Similarly, amplification of exons 12, 14, and 18 of *PDGFRA *in these GIST samples revealed clear, single bands on electrophoresis and the PCR products were directly sequenced. Three of the cases had a SNP located at base pair 49690424 A > G [GenBank: NC_006595.2] in exon 12 of *PDGFRA*. Two of the cases also had a SNPs located at base pair 49691387 A > G and 49691411 G > A [GenBank: NC_006595.2] of exon 14 of *PDGFRA *(Figure 5).

## Discussion

This study was able to ascertain *c-KIT *and *PDGFRA *mutational status of seventeen of eighteen KIT positive canine gastrointestinal stromal tumors, representing a good amplification success rate of 94% from FFPE tissues. Significantly, the study identified two distinct but overlapping mutations in exon 11 of *c-KIT *in the juxtamembrane domain. This region appears to be a mutational hotspot with an overall incidence of 35.3% in our study population of canine GISTs. The only other study of *c-KIT *mutations in canine GISTs reported mutations in two of four (50%) GISTs [3]. Human GISTs have higher incidences of *c-KIT *mutations ranging from 65% to 92% across exons 8, 9, 11, 13, and 17, a majority of which occur in the juxtamembrane domain [8,9,23,39]. In our study, no mutations were identified in exons 8, 9, 13, and 17 of *c-KIT*. None of our cases showed mutations in *PDGFRA*. Only a single amplification product was noted from the corresponding normal tissue of each GIST case, with sequencing verifying the presence of only the wild type allele in the normal tissue. These results indicate that all mutations observed arose somatically in each tumor.

Interestingly, these deletion mutations are similar to those previously found in the juxtamembrane domain of *c-KIT *in canine cutaneous mast cell tumors in our laboratory [34] and others [13]. In a previous study of 21 canine GISTs, DNA suitable for amplification was recovered from only four cases and then amplified for the KIT exon 11 of *c-KIT*, juxtamembrane domain, and sequenced [2]. Sequencing revealed mutations in two of the four canine GISTs, one with a 6 base pair deletion, TGGAAG, and insertion of CAG, predicted to translate to a deletion of tryptophan and lysine and an insertion of glycine at codon 556 [2]. This deletion is quite similar to the mutation at canine codons 556-557 discovered in the canine GISTs in our study. The second mutation discovered by Frost et al. was a substitution of T with C predicted to replace codon 575 leucine with proline [2]. We did not detect a similar mutation in our study population. The mutations observed in our study population of GISTs were clustered at codons 556-558 of *c-KIT*. No gender predilection has been reported in human GISTs, and the observation of 73% female to 27% male ratio in our study is interesting, but its significance needs further evaluation.

A simple deletion identical to the mutation at canine codons 556-557 in our study has also been reported in multiple cases of human GISTs [8,24]. In the study by Taniguchi et al., a deletion and point mutation similar to the mutation at canine codons 556-558 in our study was also detected in one of the cases they analyzed [8]. Rubin et al., found the same mutation as the deletion of canine codons 556-557 in our canine GISTs in 2 of 48 human GISTs, and they reported the same deletion of canine codons 556-558 in 1 of the 48 cases [9]. In a study of human familial GISTs, germline deletion mutations were discovered in the same region as the previously mentioned deletions [29].

All four of the SNPs found in our canine GIST samples were silent mutations, with no change predicted in the translated protein. The SNP in exon 12 of *PDGFRA *at genomic base pair 49690424 [GenBank: NC_006595.2], has been reported previously [40].

In humans, GISTs are rare neoplasms. The age-adjusted incidence of gastric mesenchymal tumors was 0.31 per 100,000 population in 2002, of which 82% were classified as GISTs [41]. The population incidence of GISTs is difficult to determine in dogs. Frost et al. commented that in dogs, gastrointestinal neoplasias account for 12-120 cases per 10,000 neoplasia cases, and in our study GISTs accounted for 39% of the total number of gastrointestinal tumors collected during the study period.

Heterozygosity with regard to mutations in the tumor sections resulted in an easily detectable aberrant banding pattern on agarose gels. While the gel electrophoresis used in this study does not resolve the normal versus the mutant alleles, which differ by only six base pairs, the normal and mutant alleles formed a heteroduplex, which contained a bubble created by the longer normal allele. The heteroduplex structure is predicted to generate a drag during gel electrophoresis giving rise to the higher band and allowing easy detection of this relatively small deletion [42]. We cannot be absolutely certain that the tumor cells are heterozygous with respect to the mutation, as the tumor sections contained some non-neoplastic components such as blood vessels. Regardless, the aberrant banding is a useful screening tool for this set of mutations.

## Conclusions

These data substantially expand the number of canine gastrointestinal stromal tumors evaluated for mutations in *c-KIT *by previous studies [2]. The mutations we have found are clustered and consistent with those shown to be activating mutations in the *c-KIT *gene of human tumors [23]. Based on these data, we can conclude that the nature of *c-KIT *mutations in GISTs in dogs is similar to that observed in humans.

The juxtamembrane domain of the KIT gene is a highly conserved region among mammals [13]. This juxtamembrane domain acts as a negative regulator of KIT activation and thus, when this particular domain is mutated, the autoinhibition is removed, allowing KIT to be activated in the absence of the KIT ligand [43]. The residues we found to be deleted in our cases of canine GISTs are the very same that Ma et al. determined to increase basal receptor phosphorylation when mutated in *c-KIT *[44]. The expression of KIT and the presence of these mutations in *c-KIT *implicate KIT in the pathogenesis of these tumors pointing to spontaneous GISTs in the dog being a relevant model for the human disease. Our results also indicate that mutations in KIT may be of prognostic and therapeutic significance in canine GISTs as they are in canine cutaneous mast cell tumors.

Numerous small molecule inhibitors that target specific tyrosine kinases, tyrosine kinase inhibitors (TKIs), have successfully been used for the treatment of human and canine cancers with mutations in KIT [45,46]. Imatinib mesylate (Gleevec, Novartis, Basel, Switzerland), has been utilized for its ability to inhibit protein tyrosine kinases since the Food and Drug Administration approved it in 2001 for the treatment of Chronic Myelogenous Leukemia (CML) [47]. Early trials demonstrated imatinib was also highly effective against GISTs [10]. Imatinib caused marked tumor response rates and dramatically increased survival times in most patients [48] and has now become the standard of care in the treatment of patients with advanced GISTs [49]. However, since with prolonged treatment clinical resistance can develop, most likely due to secondary *c-KIT *mutations, a new generation of TKIs, such as sunitinib, have been successfully introduced [48,50]. Research is ongoing to treat GISTs with resistance to imatinib and sunitinib [51]. In dogs, the TKIs Palladia (toceranib), Kinavet (masitinib), and Gleevec (imatinib) have been successfully used in numerous neoplastic diseases [45] and toceranib (Palladia, Pfizer, New York, NY, USA) and masitinib (Kinavet, AB Science, Short Hills, NJ, USA) have been registered for the use in dogs with cutaneous mast cell tumors [52-54]. In a randomized trial, dogs with KIT mutations were much more likely to respond to Palladia than those without KIT mutations [55]. To our knowledge there are no published data on the treatment of canine GISTs with TKIs. Based upon the data presented here, we propose that targeting KIT may be a rational approach to treatment of canine GISTs as well. In addition, we put forward that canine GISTs are a relevant and accessible model for human GISTs, with shared molecular pathways that can be targeted for therapy.

## Abbreviations

GIST: gastrointestinal stromal tumor; FFPE: formalin-fixed paraffin embedded; PDGFRA: platelet-derived growth factor receptor, alpha polypeptide.

## Competing interests

The authors declare that they have no competing interests.

## Authors' contributions

EGB carried out the DNA isolation, PCR, and sequence analysis. EB aided in DNA isolation, PCR, and confirmed the sequence analysis. MK and SH carried out the histopathologic evaluation and provided the KIT positive GIST samples. VYG provided laboratory resources, directed the molecular studies and supervised the sample analysis. VYG and MK planned the study and together with EGB, drafted the manuscript. All authors have read and approved the final manuscript.

## Pre-publication history

The pre-publication history for this paper can be accessed here:

http://www.biomedcentral.com/1471-2407/10/559/prepub
